# Integrating multi-omics technologies to decipher microbiome functions

**DOI:** 10.1038/s41467-026-77539-4

**Published:** 2026-09-08

**Authors:** Tim Van Den Bossche, Eunice Adeline Lazau, Velma T. E. Aho, J. Alfredo Blakeley-Ruiz, Maximilian Wolf, Benoit Josef Kunath, Luis E. Valentin-Alvarado, Patrick Hellwig, Pieter Verschaffelt, Andrew T. Rajczewski, Jannie. G. E. Henderickx, Tomi Suomi, Feng Xian, Shruti Shah, Lennart Martens, Dirk Benndorf, Samir R. Damare, Bastiaan Willem Haak, Sven-Bastiaan Haange, Paul D. Piehowski, Anne Kupczok, Daniel Figeys, Bart Mesuere, Magnus Palmblad, Robert L. Hettich, Laura L. Elo, Juan Antonio Vizcaíno, Neha Garg, Zhong Wang, Muzaffer Arıkan, Lee Ann McCue, Timothy J. Griffin, Laure-Alix Clerbaux, Robert Heyer, Marnix H. Medema, Sabine Matallana-Surget, David Gomez-Varela, Thilo Muth, Bree Tillett, Jean Armengaud, Robert D. Finn, Paul Wilmes, J. Gregory Caporaso, Lucia Grenga, Pratik Dilip Jagtap

**Affiliations:** 1 CompOmics, VIB Center for Medical Biotechnology, VIB, Ghent, Belgium; 2 Department of Biomolecular Medicine, Faculty of Medicine and Health Sciences, Ghent University, Ghent, Belgium; 3 School of Chemistry and Biochemistry, Georgia Institute of Technology, Atlanta, GA USA; 4 Faculty of Biosciences, Norwegian, University of Life Sciences, Ås, Norway; 5 Department of Plant and Microbial Biology, North Carolina State University, Raleigh, NC USA; 6 Multidimensional Omics Analyses group, Faculty of Technology, Bielefeld University, Bielefeld, Germany; 7 Graduate School DILS, Bielefeld Institute for Bioinformatics Infrastructure (BIBI), Bielefeld University, Bielefeld, Germany; 8 Bioinformatics and Artificial Intelligence, Department of Medical Informatics, Luxembourg Institute of Health (LIH), Strassen, Luxembourg; 9 Department of Biochemistry and Molecular Biology, Infection Program, Biomedicine Discovery Institute, Monash University, Clayton, VIC Australia; 10 Otto-von-Guericke-University Magdeburg, Bioprocess Engineering, Universitätsplatz 2, Magdeburg, Germany; 11 Bioprocess Engineering, Max Planck Institute for Dynamics of Complex Technical Systems, Sandtorstraße 1, Magdeburg, Germany; 12 VIB-UGent Center for Medical Biotechnology, VIB, Ghent, Belgium; 13 Department of Mathematics, Computer Science and Statistics, Ghent University, Ghent, Belgium; 14 Department of Biochemistry, Molecular Biology and Biophysics, 321 University of Minnesota, 6-155 Jackson Hall, 321 Church Street SE, University of Minnesota, Minneapolis, MN USA; 15 Center for Microbiome Analyses and Therapeutics, Leiden University Center for Infectious Diseases (LUCID), Leiden University Medical Center, Leiden, ZA the Netherlands; 16 Turku Bioscience Centre, University of Turku and Åbo Akademi University, Turku, Finland; 17 Systems Biology of Pain, Division of Pharmacology & Toxicology, Department of Pharmaceutical Sciences, Faculty of Life Sciences, University of Vienna, Vienna, Austria; 18 Biological Oceanography Division, CSIR- National Institute of Oceanography, Dona Paula, Goa India; 19 BioOrganic Mass Spectrometry Laboratory (LSMBO), IPHC UMR7178, CNRS, Université de Strasbourg, Strasbourg, France; 20 Infrastructure Nationale de Protéomique ProFI-UAR, Strasbourg, France; 21 Applied Biosciences and Process Engineering, Anhalt University of Applied Sciences, Bernburger Str. 55, Köthen, Germany; 22 Center for Infection and Molecular Medicine, Amsterdam UMC, Amsterdam, The Netherlands; 23 Department of Molecular Toxicology, Helmholtz Centre for Environmental Research – UFZ, Permoserstr. 15, Leipzig, Germany; 24 Environmental and Molecular Sciences Laboratory, Pacific Northwest National Laboratory, Richland, WA USA; 25 Bioinformatics Group, Wageningen University & Research, Wageningen, The Netherlands; 26 Quadram Institute Bioscience, Norwich Research Park, Norwich, Norfolk, UK, University of East Anglia, Norwich, Norfolk UK; 27 Center for Proteomics and Metabolomics, Leiden University Medical Center, Leiden, RC The Netherlands; 28 Biosciences Division, Oak RIdge National Laboratory, Oak Ridge, TN USA; 29 Institute of Biomedicine, University of Turku, Turku, Finland; 30 European Molecular Biology Laboratory - European Bioinformatics Institute (EMBL-EBI), Wellcome Genome Campus, Hinxton, Cambridge, UK; 31 School of Chemistry and Biochemistry, Center for Microbial Dynamics and Infection, Georgia Institute of Technology, Atlanta, GA USA; 32 Department of Energy Joint Genome Institute, Lawrence Berkeley National Laboratory, 1 Cyclotron Road, Berkeley, CA USA; 33 School of Natural Sciences, University of California at Merced, 5200 Lake Rd, Merced, CA USA; 34 Environmental Genomics and Systems Biology Division, Lawrence Berkeley National Laboratory, 1 Cyclotron Road, Berkeley, CA USA; 35 Biotechnology Division, Department of Biology, Faculty of Science, Istanbul University, Istanbul, Türkiye; 36 Biological Sciences Division, Pacific Northwest National Laboratory, Richland, WA USA; 37 Laboratory of Hepato-Gastroenterology, Institute of Experimental and Clinical Research, UCLouvain, Brussels Belgium; 38 Leibniz-Institut für Analytische Wissenschaften – ISAS – e.V., Dortmund, Germany; 39 Biofonderie de l’Alliance Sorbonne Université, UAR 2037, Sorbonne Université, CNRS, Université de Technologie de Compiègne, 75005, Paris, Cedex France; 40 International Research Hub for Marine Pollutions CIC, Technology House, Glasgow, UK; 41 Center of Excellence for Metaproteomics University of Vienna - Bruker Daltonics, University of Vienna, Vienna, Austria; 42 Data Competence Center MF 2, Robert Koch Institute, Berlin, Germany; 43 Frazer Institute, The University of Queensland, Woolloongabba, Brisbane, Queensland Australia; 44 Département Médicaments et Technologies pour la Santé (DMTS), Université Paris-Saclay, CEA, INRAE, SPI, Bagnols-sur-Cèze, France; 45 Luxembourg Centre for Systems Biomedicine, University of Luxembourg, Esch-sur-Alzette, Luxembourg and Department of Life Sciences and Medicine, University of Luxembourg, Esch-sur-Alzette, Luxembourg; 46 Pathogen and Microbiome Institute, Northern Arizona University, Flagstaff, AZ USA; 47 Infrastructure Nationale de Protéomique, ProFI, UAR 2048, Bagnols-sur-Cèze, France

**Keywords:** Microbiome, Microbial ecology

## Abstract

Multi-omics approaches have revolutionized our understanding of microbial communities by enabling simultaneous interrogation of genomic, transcriptomic, proteomic, and metabolomic data. The systematic integration and analysis of these deep datasets help decipher the functional roles of microbiomes, providing critical insights into microbial activities, interactions, and dynamics across diverse environments. Biological complexity makes multi-omics analysis of a single, isolated organism demanding but highly informative, yet this complexity increases further when samples comprise hundreds to thousands of individual species. As microbiome research continues to expand into clinical, environmental, and engineered systems, standardized workflows, benchmarked datasets, and community-driven initiatives are essential to ensure reproducibility, standardization and interpretability. Establishing and disseminating best practices for experimental design, data processing, and integrative analyses will be critical for maximizing comparability and scientific rigor across studies. This perspective highlights recent advances in multi-omics microbiome research, outlines key obstacles in data integration and metadata harmonization, and proposes a collaborative roadmap for scalable, FAIR-compliant multi-omics investigations and potentially disruptive Artificial Intelligence (AI) advances comparable to those of AlphaFold in the field of microbiome science.

## Abstract (web summary)

In this Perspective, the authors discuss recent advances in multi-omics microbiome research, outlining key obstacles in data integration and metadata harmonization, and proposing a roadmap for scalable, FAIR-compliant multi-omics investigations and potentially disruptive Artificial Intelligence advances.

## Introduction

Multi‑omics technologies increasingly provide insight into microbiome function across environmental, engineered, and host‑associated systems. Integrating metagenomics, metatranscriptomics, metaproteomics, and metabolomics links microbial composition to activity and ecological interactions^1,2^. These approaches extend functional interpretation beyond marker‑gene surveys, enabling the identification of novel taxa, biosynthetic pathways, and biochemical functions, offering a route to unravel previously inaccessible mechanisms and propose new hypotheses for causal relationships between microbiome shifts and host or environmental phenotypes. These multi‑layered datasets underpin emerging applications in precision therapeutics, sustainable agriculture, and ecosystem stewardship, reinforcing a “One Health” perspective that connects microbiome function across human, animal, and environmental domains^3^.

Despite major advances in standardized profiling of taxa and genomic potential, robust and scalable integration frameworks are still needed to bridge genotype with phenotype and resolve microbe–microbe and host–microbe interactions in complex communities. Several recent reviews summarize the current state-of-the-art, opportunities and challenges of multi-omics microbiome research from different perspectives including methodological developments and analytical principles, host–microbiome integration, and applications in specific biological or clinical contexts^1,4–7^. Yet as the field embraces multi‑omics integration, a proliferation of analytical strategies has produced heterogeneous, non‑standardized outputs that are difficult to compare or synthesize across studies. Consequently, the central bottleneck is shifting from reproducible data generation toward reproducible interpretation. Against this backdrop, coordinated international and interdisciplinary efforts are urgently needed to benchmark methods, harmonize data and metadata, and generate interoperable datasets. Rather than serving as an exhaustive review of every subfield, this Perspective provides an educational overview of the current landscape together with an operational roadmap for researchers across career stages and areas of omics expertise and interdisciplinary teams developing integrated multi-omics microbiome studies. The primary audience is investigators or teams designing or interpreting microbiome multi-omics studies, whether they are entering the field or already established within a single omics domain. Community-level recommendations are included because implementation of good practice at the individual-study level depends on enabling infrastructure, including interoperable repositories, standards, shared reference materials, and benchmarking resources. By bringing together experimental, computational, interpretive, standardization, and translational perspectives, we argue that multi-omics studies should be evaluated by the incremental biological information produced by integration rather than by the number of molecular layers measured, around a framework of reproducible and benchmarkable interpretation. This Perspective integrates three linked elements: (i) a discussion of the key strengths, limitations, and recurring challenges of major omics technologies from both experimental and computational viewpoints; (ii) an overview of the benefits and applications of integrated multi‑omics approaches in microbiome research; and (iii) an expert‑informed roadmap outlining best practices, future priorities, and areas where improved standards are needed. This framing complements existing reviews by moving from a description of multi-omics capabilities toward providing an operational roadmap for interpretable, comparable, and AI-ready microbiome multi-omics data.

### Strengths and challenges of individual omics approaches in integrated microbiome research

Multi‑omics approaches integrate complementary molecular layers to resolve microbiome function with greater precision. Metagenomics, specifically from whole genome shotgun sequencing and reconstruction of Metagenome-Assembled Genomes (MAGs), defines community membership and genetic potential, including previously undescribed lineages^8,9^. Metatranscriptomics identifies actively transcribed genes and eukaryotic gene structures^10^. Metaproteomics detects and quantifies proteins to reveal executed functions, active pathways, and host proteome shifts^11^. Metabolomics profiles small molecules that reflect enzymatic activity and mediate microbe–microbe and microbe–host interactions^12^. Each omics data type contributes valuable biological insight, despite challenges associated with generating and analyzing these data that can introduce significant variability and hinder reproducibility, a challenge well-documented previously^13^. The strengths and limitations of each omics layer are summarized in Table 1.

**Table 1:** Strengths, challenges, and solutions of each omics layer

Omics layer	Strengths	Challenges	Potential solutions
**Metagenomics**^**§**^	· Identification of functional genes · Genome-resolved analysis · Species- and strain-level resolution · High scalability	· Genes/genomes can be fragmented, thus rendering comprehensive MAG assembly difficult, especially for microbial eukaryotic genomes · MAG contamination · Gene prediction may contain inaccuracies · Extraction of high molecular weight (HMW) DNA from complex matrices (e.g., soil) is difficult but required for long-read sequencing	· High coverage, long-read sequencing, co-assembly and co-binning are strategies to increase assembly contiguity, reduce MAG contamination and enhance the representation of low-abundance taxa · Validate gene predictions with complementary omics layers · Use matrix-specific HMW DNA extraction protocols tailored to remove inhibitors (e.g., humic acids)
**Metatranscriptomics**	· Capture of actively transcribed genes · Improve identification of small genes and eukaryotic transcripts · Detect rapid cellular responses	· Transcript abundance can be underestimated because RNA is unstable · Samples are often dominated by rRNA, which can bias against low abundance transcripts if sequencing depth is shallow · Transcript level changes may not predict protein abundance changes · Low abundant transcripts can be difficult to assemble	· Rapid processing with stabilization buffers is required · rRNA depletion can reduce bias and enable less abundant transcript detection · Independent observations using complementary omics layers alleviated prediction bias · Co-assembly with metagenomics or MAG-based mapping improves assembly and cross-layer integration
**Metaproteomics**	· Identification of active functions · Taxon-resolved peptide mapping · Post-translational modification detection · Advanced techniques including stable isotope probing (SIP and bioorthogonal non-canonical amino acid tagging or threonine-derived non-canonical amino acid tagging (BONCAT/THRONCAT) can detect newly synthesized proteins and enhance their detection with click chemistry	· Many peptide sequences are not unique to just one protein or organism, (i.e. protein inference problem) making source/organism attribution difficult · Dependence on assembly and gene annotation quality for peptide and protein identification · Substantial missingness in the detection of peptide-spectrum matches (PSMs) · Protein detection does not necessarily reflect enzymatic activity · Quantitative reproducibility and proteome coverage can be highly variable	· Can be minimized with unique peptide filtering and/or clustering approaches · Curated reference databases, especially a metagenome from the same sample yields optimal peptide identification · AI-based rescoring approaches can recover missed low-abundance PSMs · Advanced differential analysis approaches can deal with substantial missingness · Post-translational modifications and click chemistry analyses can provide more information on functional states · Data-Independent Acquisition analysis approaches can increase proteome coverage
**Metabolomics**	· Direct evidence of biochemical activity · Detection of metabolites from hosts and microbes · Targeted metabolite approaches link to genome-encoded pathways · Untargeted approaches enable broad discovery	· Compound annotation rates are low · Source/organism attribution is difficult · Some metabolites can be derived from and feed into multiple pathways and can be readily transported, complicating data interpretation · The chemical diversity of metabolites limits the effectiveness of a single extraction method and complicates integration	· Molecular networking and experimental (or in silico) spectral libraries can improve compound identification and focusing on targeted subsets can improve validation · Stable isotope probing and matching against source-specific databases can enhance source attribution · Careful interpretation and multi-omics comparison with transcripts and protein levels can generate confidence in pathway analysis · Use complementary liquid chromatography separations (hydrophilic and hydrophobic) and multiple MS ionization modes (positive and negative)

^§^ Throughout this work, metagenomics references whole genome shotgun metagenomics (exclusive of amplicon sequencing approaches).

### Benefits and applications of multi-omics

**Fig. 1:**
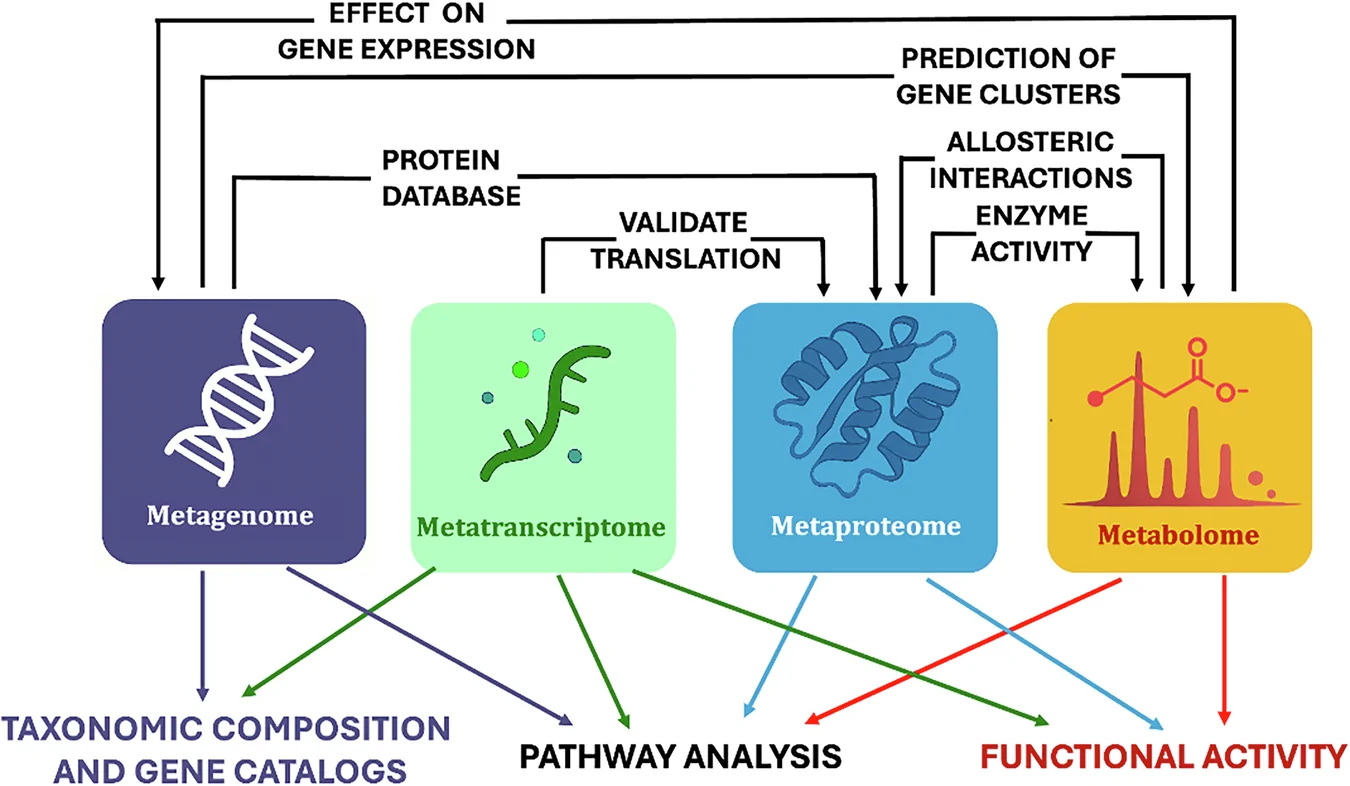
Molecular interactions are complex and non-linear. Integrating multi-omics data across metagenomics, metatranscriptomics, metaproteomics, and metabolomics can aid in interpretation, provide supporting evidence or predict novel interactions (Table 2 provides representative selection of published multi-omics studies). Prototypical examples of integration include the following. The read-, contig-, or MAG-based gene catalogs from metagenomics support species- and strain-resolved analyses and serve as shared references for metatranscriptomics and metaproteomics, while also linking to metabolomics through pathway-level interpretation. Metatranscriptomics maps to metagenomic assemblies and integrates with metaproteomics for functional validation and connects to metabolomics through pathway analysis. Coupling metagenomics with metatranscriptomics identifies which genes are actively transcribed and improves gene prediction, thereby enhancing functional annotation^71^. Metaproteomics typically relies on MAG-derived protein databases^72^ for taxon-resolved peptide mapping, provides functional validation of metabolomic pathways, and confirms whether detected transcripts are translated. Incorporating metabolomics adds direct biochemical evidence, confirming metabolic shifts inferred from proteomic profiles and illuminating host–microbe chemical cross‑talk, including metabolites that can be linked to actionable interventions capable of reshaping ecosystem balance^73^. The upper arrows are some examples of how each omics layer can aid interpretation of additional omics layers. The lower arrows highlight each omics layer’s contribution to common analyses.

Multi-omics integration extends microbiome analysis beyond community composition and predicted gene content. Figure 1 illustrates some of the fundamental interactions that have been studied using multi‑omics data to connect patterns across molecular layers and reveal dynamic, context-dependent biological responses to changing conditions. Together, these layers capture the molecular flow from potential to function, each responding on distinct timescales: the transcriptome reflects rapid, often transient responses; metaproteomic profiles reveal downstream adjustments in cellular machinery; and metabolomic changes mark the resulting biochemical activity. Because of this significant temporal decoupling between when a gene is transcribed and when the resulting protein or metabolite trait is observable, linking gene expression directly to a phenotypic outcome from a single snapshot is inherently difficult. Consequently, sampling at multiple time points is essential. Time-series (longitudinal), paired (each omics layer derived from the same biological material) multi-omics allows researchers to capture the dynamic cascade of expression, resolve translational delays, and more accurately map genetic potential to executed functions and phenotypic traits^4,14^. Crucially, information flow in multi‑omics networks is not unidirectional. Metabolites modulate protein activity through allosteric interactions and post‑translational modifications, while both metabolites and proteins influence DNA and RNA function—for example, metabolite levels can regulate gene‑regulatory protein binding or activate RNA riboswitches that reshape transcriptional programs^15^. Biological signals therefore emerge from interactions across layers rather than from any single omics modality alone.

Recent large studies have increasingly taken advantage of multi-omics integration to provide unique insights into microbiome functions across ecological and clinical contexts (summarized in Table 2). However, it is important to emphasize that use of multi‑omics is most powerful when deployed as a fit‑for‑purpose strategy rather than a layer‑counting exercise. For example, metagenomics alone is appropriate when the study aims to resolve community composition and when taxonomic structure itself carries the relevant signal^16–18^. The value of multi-omics integration lies in how well the chosen omics layers align with the biological question, the sample type, and the decision‑making context. Case studies across microbiome research consistently show that effective integration depends on prioritizing measured functional activity over genetic potential, elevating mechanistic insight above descriptive associations, and clearly separating taxonomic signals from functional ones. While integrating multiple omics layers can greatly enhance mechanistic and causal understanding in microbiome research, these approaches are undeniably resource‑intensive. It is therefore both reasonable and necessary to consider cost as a key factor when designing a multi‑omics study, ensuring that the chosen omics layers align with the study’s scientific priorities and practical constraints. Moreover, it is worth noting that adding multiple layers can lead to recognized challenges such as extensive, non-aligned missingness across data layers, imperfect sample matching, cross‑platform batch effects that obscure biological structure, and analyses limited by incompletely curated databases. Finally, multi-omics approaches can generate apparent discrepancies when layers are compared in isolation rather than truly integrated (*e.g*., function appearing upregulated at the RNA level while downregulated at the protein level). Such discrepancies often reflect genuine biological phenomena rather than technical artefacts but interpreting them requires a clear understanding of what each molecular layer captures and its relevance to the biological question at hand. Selecting the appropriate level of integration from the outset is therefore essential to avoid misinterpretation.

**Table 2:** Non-exhaustive overview of notable multi-omics studies combining three or more omics layers

Case Study	MG	MT	MP	MM	Highlights	Reference
“Lunar Palace 365” experiment	103	–	90	56	Specific gut microbes can positively influence mood and mental health in long-term closed environments by modulating neuroactive compounds and metabolic pathways.	^74^
**Biological wastewater treatment plant metaomics**	53	53	53	53	Microbial communities in wastewater treatment adapt to disturbances through phenotypic flexibility and complementary ecological roles.	^75^
Oral-gut axis microbiome in familial type 1 diabetes	158	138	71	–	Strain level analysis of oral-gut axis in type 1 diabetes mellitus and impact of the oral microbiota’s alteration on the gut microbiome.	^76^
Dental Plaque from Patients with Diabetes and Periodontal Disease	–	–	97	97	A multi-omic analysis of dental plaque revealed distinct microbial, lipid, and protein signatures associated with prediabetes, type 2 diabetes, and periodontal disease, including a novel lipid biosynthesis pathway.	^77^
Xanthan gum-degrading gut microbiome	✔	✔	✔	✔	Discovery of a xanthan gum food chain in the human gut microbiome is contingent on a single uncultured Ruminococcaceae bacterium.	^78^
Activated sludge from wastewater treatment plant for organic micropollutant degradation	✔	–	✔	✔	Multi-omic analysis connecting OMP functional groups, degradation enzymes, metabolic pathways, and microbial phyla reveal Actinobacteria, Bacteroidetes, and Proteobacteria as primary contributors.	^79^
Gnotobiotic mice colonized with age- and ponderal growth-associated gut bacterial strains	✔	✔	–	✔	*P. copri* metabolism of MDCF-2 glycans and interactions with other microbes including *Bifidobacterium infantis* reveal structure-function relationships with implications for malnourishment therapies.	^80^
Longitudinal IBDMDB cohort of 132 controls and patients with IBD	1036	440	–	508	Inactivation of IBS drug 5-ASA is associated with 12 previously uncharacterized microbial acetyltransferases.	^81^
**Stool of IBD patients and healthy controls**	182	–	182	182	Crohn’s disease subtypes are distinguishable by MP and MM but not microbiome MG or host genetics.	^82^
Vietnamese rice paddy soil derived cultures	24	24	12	–	Isolation of *Paraclostridium* sp. strain EML, confirmed to actively anaerobically methylate arsenic.	^83^
Rumen microbiome of dairy cows	9	9	–	9	Identification of *Selenomonas* and Succinivibrionaceae keystone members in the rumen of high-efficiency animals and six differentiating metabolites.	^84^
**Active DNA viruses and RNA viruses in a native prairie soil**	2	12	12	–	Extreme shifts in soil moisture have dramatic impacts on the composition, activity and potential functions of both DNA and RNA soil viruses.	^85^
Fecal and serum samples from ulcerative colitis patients and controls	250	–	250	250	Bacteroides vulgatus *produce overabundance of proteases in UC patients; use of a broad-spectrum protease inhibitor improved B. vulgatus-induced barrier dysfunction.*	^86^

Bolded entries correspond to studies described in the main text. MG = metagenomics; MT = metatranscriptomics; MP = metaproteomics; MM = metabolomics. Numbers indicate the reported sample size for each omics layer. Check marks denote studies where sample size was not explicitly stated.

The wastewater bioreactor study by Herold et al.  is an excellent example of matched omics data integration, going beyond omic association based on statistical inference. Here, a 14-month weekly in situ time series was complemented by deep sequencing of 12 ex situ bioreactor samples to directly map dynamic microbial responses to perturbations. Metagenomics alone outlined the fundamental niches of reconstructed populations, yet the addition of metatranscriptomics, metaproteomics, and metabolomics revealed the phenotypic plasticity and niche complementarity that stabilized reactor performance^14^. A severe substrate-level disturbance altered community composition and gene expression, followed by recovery of individual populations within ten sludge age cycles; complementary ex situ pulse experiments showed transcriptional responses within five hours, with *Microthrix* illustrating a distinct phenotypic-heterogeneity strategy. These dynamics underscore how multi‑omics captures both acute perturbation responses and the ecological mechanisms that underpin resilience.

In complex host-dominated systems, where disease-associated microbiome changes can be subtle, each additional layer of a multi-omic study can provide greater resolution to classify disease states for biomarker discovery. Gonzalez et al. showed that neither metagenomics nor host genetics could differentiate ileal from colonic Crohn’s disease, whereas metabolomics, metaproteomics, and integrated multi‑omic signatures separated the two subtypes^19^. Colonic disease exhibited neutrophil‑associated protein enrichment and associations with *Bacteroides vulgatus*, while ileal disease was characterized by elevated bile acids, depletion of *Faecalibacterium prausnitzii*, and enrichment of bile‑acid‑adapted taxa. These activity‑based readouts enabled the identification of location‑specific protein-ratio biomarker pairs that outperformed the standard test for gut inflammation, e.g. fecal calprotectin, illustrating the translational value of multi‑omics for precision diagnostics.

Environmental virome studies further highlight the ability of multi‑omics to reveal active ecological processes that are otherwise cryptic. In prairie soils, Wu et al. integrated metagenomics, metatranscriptomics and metaproteomics to identify active DNA and RNA viruses, quantify their responses to moisture variation, and validate translation of viral transcripts, including phylogenetically distinct chaperonins^20^. These findings emphasize that although currently undercharacterized, viral populations are diverse, responsive to relevant environmental shifts, and functionally consequential—features that become visible only through multi‑omic integration.

Across these examples, the contribution of additional omics layers depended strongly on the biological question, ecosystem complexity, and type of functional signal being investigated. In the dynamic wastewater system, transcript, protein, and metabolite data collected via coordinated temporal measurements connected community shifts to phenotypic plasticity, niche complementarity, and resilience while distinguishing transient transcriptional changes from sustained functional adaptation. In Crohn’s disease, metaproteomic and metabolomic profiles distinguished ileal and colonic disease profiles that were not resolved by metagenomics or host genetics alone. This shows how mass-spectrometry-based layers provided discriminatory power beyond sequencing-based measurements alone by resolving activity-linked molecular signatures associated with disease state and phenotype. In environmentally under-characterized systems such as the prairie soil virome, integration primarily improved the detection and validation of previously unresolved biological entities and functions by leveraging cross-layer support between sequencing- and mass-spectrometry-derived data. Together, these examples show that the value of multi-omics lies not in the number of molecular layers generated, but in whether integration changes the inferred relationships between microbial communities, function, and phenotype.

### Common pitfalls limiting interpretable microbiome multi-omics

Despite major advances in microbiome multi-omics, several recurring practices continue to limit cross-study comparability and hamper interpretation. Many of these limitations arise not from insufficient molecular resolution, but from difficulties in generating reproducible cross-layer inference. A common misconception is that increasing the number of omics layers necessarily improves interpretation. Additional layers are primarily valuable when they resolve processes that cannot be inferred from simpler measurements alone, rather than serving as parallel descriptive datasets. Underpowered multi-omics studies with unmatched sampling or poorly coordinated acquisition frequently generate integration artifacts that are difficult to distinguish from biological signals. Coordinated variation across genes, transcripts, proteins, metabolites, or taxa is frequently interpreted as evidence of mechanistic linkage. However, correlations across molecular layers may arise from indirect ecological effects, shared environmental drivers, or compositional dependencies rather than direct biochemical interactions. This issue is particularly important in microbiome datasets, where relative abundance constraints can generate spurious correlations between features even in the absence of direct biological association^21^. As a result, statistical integration alone rarely provides sufficient evidence for mechanism, underscoring the importance of perturbation experiments, longitudinal sampling, and targeted validation strategies. Additionally, functional interpretation remains strongly constrained by the structure and coverage of current reference databases. Annotation performance varies substantially across ecosystems and phylogenetic groups, with environmentally diverse microbiomes frequently containing large unresolved fractions. For example, environmentally derived metaproteomic and metabolomic datasets often contain large numbers of unmatched spectra or unannotated features^22,23^, limiting downstream inference even when molecular signals are reproducibly detected. These constraints are not unique to any single layer: sequencing-based inference is limited by ambiguities in reading-frame assignment and genetic-code usage, particularly in Archaea and phages, as well as by incomplete gene models, whereas mass-spectrometry-based layers are affected by matrix-dependent ionization and ion suppression. Metaproteomics is further constrained by unanticipated post-translational modifications and the protein inference problem^24,25^. For metabolomics, variations in extraction chemistry, chromatographic methods, and matrix effects can selectively bias metabolite classes recovered from complex microbial communities. Untargeted omics measurements offer increasingly in-depth coverage of transcripts, proteins or metabolites; however, these approaches rely on relative quantification which necessitates stringent control of experimental parameters to ensure accurate comparisons across samples within a study. Even with standardized protocols, small differences in buffer pH, incubation times, reagent batches or mass spectrometry response can result in batch effects that can mask the true biological signals^26,27^.

Consequently, apparent differences in functional profiles may partly reflect differences in annotation coverage and database composition rather than underlying biological variation alone. This limitation is particularly important when comparing datasets generated using different annotation frameworks or reference systems. Despite widespread claims that multi-omics improves functional interpretation, the contribution of individual molecular layers is rarely evaluated explicitly. In many studies, it remains unclear whether additional omics measurements substantially altered biological conclusions relative to other lower-complexity approaches.

Together, these issues highlight that the central challenge in microbiome multi-omics is no longer simply generating increasingly complex datasets but determining how additional molecular measurements improve biological interpretation in a reproducible and comparable manner.

### Solutions and best practices for advancing multi‑omics integration

Integrating multi-omics data in microbiome research requires coordination across experimental design, sample processing, metadata annotation, and computational analysis. Challenges that affect individual omics domains, including sparsity, compositionality, dynamic range limitations, and incomplete reference coverage, become amplified during cross-omics integration, particularly in microbiome systems characterized by high biological and environmental variability^28^. These constraints complicate cross-layer comparison and increase the risk of technical and analytical inconsistencies. Robust integration therefore depends on coordinated sampling strategies, harmonized metadata collection, interoperable data formats and identifiers for genes, proteins and small molecules, and reproducible computational workflows. Whenever possible, DNA, RNA, proteins, and metabolites should be derived from the same biological aliquot to preserve cross-layer correspondence and minimize technical variability^28,29^.

In host-associated microbiome studies, these requirements become even more demanding due to the need to align microbial and host-derived molecular measurements within consistent experimental and computational frameworks^6^. Unraveling previously inaccessible mechanisms and causal relationships between microbiome shifts and host or environmental phenotypes requires incorporating as much phenotyping information as possible. This provides the necessary anchor to interpret microbial function in vivo. While metagenomics, metatranscriptomics, and metabolomics capture community structure and activity, they remain inferential without corresponding readouts from the host or environment that is being studied. Phenotypes such as health state, respiratory function in cystic fibrosis, or a relevant functional endpoint of a bioreactor, serve as the functional outcomes that define biological relevance. These measurements enable discrimination between microbiome features that are mechanistically impactful versus epiphenomenal. Interestingly, host phenotypes provide temporal resolution for causality when integrated in longitudinal designs. Thus, multi-omics layers generate mechanistic hypotheses, while host or environmental phenotyping constrains and validates them. In this framework, phenotype serves as the system-level ground truth linking microbiome activity to host physiology and clinical outcome or ecosystem service and biogeochemical outcomes.

Uneven molecular coverage across omics layers remains another major operational challenge. Sequencing-based approaches routinely achieve broad representation of microbial communities, whereas metaproteomics and metabolomics remain constrained by peptide detectability, chemical diversity, dynamic range limitations, and incomplete reference libraries^30^. Metabolomics is even more restricted: no single platform captures the full chemical diversity of the metabolome, and annotation remains limited by the lack of reference standards. These disparities hinder integrative analyses, can distort cross-layer inference, and can bias downstream interpretation if modality-specific limitations are not explicitly considered. Nonetheless, continued improvements in mass spectrometry sensitivity, coverage, and quantification are progressively reducing some of these acquisition-related limitations^31,32^.

**Fig. 2:**
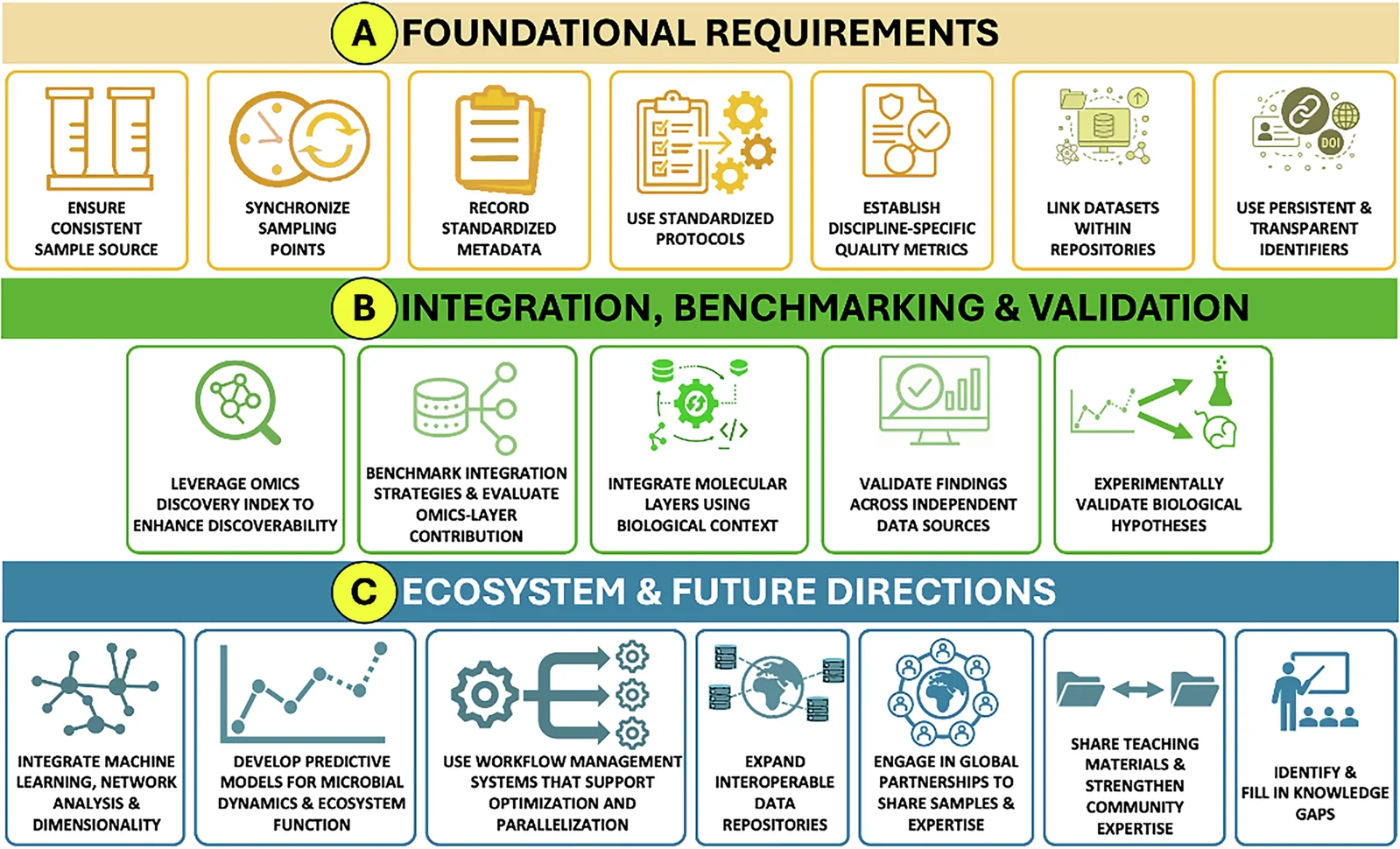
Best practices and recommendations for advancing multi‑omics integration. Recommendations are grouped into three categories: **A** Foundational requirements: study-design and data-generation practices ensuring cross-layer comparability (sample sourcing, sampling synchronization, metadata, protocols, quality metrics, dataset linkage, persistent identifiers). **B** Integration, Benchmarking and validation: computational practices for combining and benchmarking multi-omics data (standardized reporting templates, benchmarking against reference datasets, OmicsDI, cross-layer linkage, independent validation, in vitro/in vivo model comparison). **C** Ecosystem and future directions: community infrastructure sustaining integration over time (machine learning and predictive modeling, workflow management systems, data repositories, global partnerships, education, and knowledge-gap identification).

Addressing these challenges will require coordinated community-driven strategies spanning study design, metadata harmonization, quality control, interoperability standards, and reproducible workflow development. The following recommendations synthesize current best practices (Fig. 2).

### Foundational requirements

Effective microbiome multi‑omics integration begins with coordinated study design that ensures direct correspondence across molecular layers. Using a shared source sample across genomics, transcriptomics, proteomics, and metabolomics reduces technical variability^28^ and enables robust cross‑layer mapping. Time‑aligned sampling with consistent biomass further minimizes variance while accommodating biological lag across omics layers^23,26^. Comprehensive, standardized metadata capture such as sample attributes, experimental conditions, processing steps, and analytical provenance is essential for reproducibility and cross‑study comparability. Existing community metadata standard formats should be used for maximal interoperability of the metadata (MIXS for metagenomics/metatranscriptomics; SDRF for metaproteomics; and METAS for metabolomics). Quality control (QC) underpins all multi‑omics workflows. Harmonized extraction protocols, quantitative internal standards, contamination‑prevention practices, and instrument‑performance monitoring ensure that relative and absolute measurements remain comparable across samples and studies. Since extraction efficiency differs between Gram-positive and Gram-negative bacteria, optimizing extraction methods is recommended to reduce Gram-based extraction bias while preserving the omics molecules of interest. Instrument performance should be assessed using appropriate QC samples between each analysis block to ensure consistent analytical performance^33^. Relevant design information such as processing and analysis blocks, run order, and analysis dates should be captured in the metadata to inform any downstream batch effect corrections. For metabolomics, the extreme chemical and structural diversity of microbial metabolites demands stringent control of sample handling, including rapid quenching, storage, and extraction workflows. When these factors are optimized, deep metabolomics can be integrated with genomic and transcriptomic layers to more accurately link microbial phenotype to the underlying biochemical activities encoded in the microbiome. Provenance tracking including workflow parameters, software versions, run order, and analysis blocks supports transparency and enables downstream correction of batch effects. Capturing this information in structured metadata is necessary for FAIR‑aligned data reuse. Finally, persistent identifiers (e.g., UUIDs for the same biological material^34^, BioSamples^35,36^ accessions, and the use of interoperable gene, protein and small molecule identifiers) and standardized formats (MIxS, SDRF‑Proteomics^37^, SMetaS^38^) ensure that samples, features, and datasets can be reliably linked across repositories and analytical layers. These identifiers reduce manual curation errors and maintain traceability across workflows. However, harmonizing identifiers remains challenging because database accessions, locus tags, gene and protein names, and organism-specific nomenclatures often coexist, highlighting the need for interoperable cross-references across repositories and publications.

### Integration, benchmarking & validation

Interoperability is the backbone of multi‑omics integration. Interoperability in microbiome multi‑omics integration refers to the exchange, linkage, and joint interpretation of data across genomic, transcriptomic, proteomic, and metabolomic layers. Despite progress, major barriers persist in siloed domain‑specific repositories (ENA/GenBank, GEO, PRIDE/ProteomeXchange, MetaboLights, GNPS/MassIVE)^39,40^. Aggregators such as OmicsDI can, however, greatly improve discoverability by linking datasets through shared PMIDs and harmonized metadata views^41^. Benchmarking is essential for evaluating integration strategies. Synthetic communities (recently also renamed as ‘defined microbial communities’^42^ and controlled models can provide standardized testbeds for workflow development and bridge the gap between complex natural systems and tractable experimental designs^43^. Reproducibility of synthetic community-based benchmarking depends on the provenance and accessibility of community members. Hence, authenticated strains should be deposited in established culture collections such as DSMZ or ATCC with accessions and abundance ratios reported in sufficient detail for reconstruction, as recently proposed for plant–microbiota research^44^. Researchers are encouraged, wherever possible, to assemble synthetic communities from established culture collections. Interlaboratory studies, or ring trials, in which the same reference material is distributed across participating laboratories, further allow technical variance to be separated from biological effect and provide an empirical basis for harmonized SOPs and performance criteria^45,46^. Benchmarking with synthetic datasets further strengthens data quality. We encourage the scientific community to establish a structured suite of well-defined, domain-relevant benchmark tasks. These should include clearly specified prediction targets spanning diverse ecosystems (e.g., host-associated, soil, marine), such as taxonomic composition from multi-omics inputs, metabolic pathway activity, host phenotype association, ecosystem stability under perturbation, or longitudinal state transitions. Each benchmark task should include clearly specified inputs, outputs, training and test splits, baseline models, and agreed-upon evaluation metrics: i) combining predictive performance (e.g., cross-validated accuracy, calibration and uncertainty estimation), ii) robustness (e.g., across cohorts), iii) resistance to data leakage (including separation of training and test sets by subject, site, time series, or study) and iv) biological plausibility (e.g., consistency with known pathways, and iterative wet-lab experimental validation). Importantly, benchmarks should be tiered, progressing from simpler settings, such as predicting taxonomy, functional profiles, or pathway activity from single-omics inputs, to more complex challenges, including multi-omics integration, assessment of the contribution and added biological value of transcriptomic, proteomic, or metabolomic layers, perturbation-response prediction, causal inference, and temporal forecasting of microbiome state transitions. Typically, they should be constructed from datasets that are already available or via generation of specific multi-omics benchmarking data that would be realistically attainable in the near term, complemented with appropriate measurements of phenotypic characteristics. Beyond predictive performance, findings should be validated across independent datasets, biological matrices, and experimental models. Validation across matrices and cohorts can test the generalizability and biological relevance of observed associations, while comparison with external reference resources can reveal annotation gaps and systematic biases. Mechanistic hypotheses derived from multi-omics integration should subsequently be experimentally validated using appropriate in vitro or in vivo models.

To support this effort, we propose the establishment of community-driven consortia tasked with creating, assembling, curating, and maintaining these benchmarks. Such consortia could coordinate the aggregation of existing public datasets, harmonize metadata and preprocessing pipelines, and define standardized data to ensure fair comparisons. Special attention should be given to representativeness, by including diverse populations for host-microbiota cohorts, ecological contexts, and experimental conditions, and by explicitly documenting potential biases or gaps. Governance models could include open calls for dataset contributions, periodic community challenges, and versioned benchmark releases, allowing continuous refinement as new data and methods emerge. Ultimately, such coordinated efforts would transform fragmented datasets into robust reference standards for benchmarking next-generation artificial intelligence (AI) methods in microbiome research. AI approaches such as machine learning (ML) and deep learning (DL) hold the potential to accelerate our understanding of microbiome function (e.g., by functional prediction of sequences^47^) and to enable the engineering of microbes and microbiomes for biomedical and biotechnological advances. However, rather than treating AI as a general promise, a benchmark-driven framework would clarify which biological questions are tractable, which omics layers provide added, measurable value, and which models generalize across cohorts and ecosystems. For example, benchmark datasets could evaluate whether transcriptomic, proteomic, or metabolomic measurements improve prediction of microbiome responses to perturbations relative to metagenomics alone. Comparing model performance across matched multi-omics datasets, including analyses in which specific layers are systematically removed, would help determine when additional molecular measurements provide reproducible added value across cohorts or ecosystems. Realizing the full potential of AI in microbiome science will therefore depend on sustained coordination across omics communities, shared standards for benchmark construction, and an explicit commitment to FAIR, representative, and experimentally grounded reference datasets.

### Ecosystem and future directions

The future of microbiome multi‑omics integration depends on a coordinated ecosystem spanning data generation, repositories, computational infrastructure, and community‑driven standards. AI methods (ML/DL) hold transformative potential for predicting microbial function, modeling ecosystem dynamics, and engineering microbiomes. However, progress depends on generating high‑quality, standardized, FAIR multi‑omics datasets suitable for training robust models. Breakthroughs comparable to AlphaFold will require representative, benchmark‑ready datasets with explicit evaluation metrics and safeguards against data leakage. Repositories must evolve to support cross‑omics discoverability, standardized metadata, and persistent identifiers. They should also capture underrepresented microbial functions and regional diversity to improve global representation. Workflows and computational infrastructure must address scalability, missing data, sparse feature spaces, and heterogeneous inputs. End‑to‑end platforms (QIIME 2^48^, bioBakery 3^49^, gNOMO2^50^, IMP^51^, MGnify^52^) and statistical integration frameworks (MOFA^53^, mixOmics^54^, miBiOmics^55^, mmvec^56^) offer complementary approaches. Workflow optimization, parallelization, and access to high‑performance computing remain essential, but disparities in computational expertise must be addressed through community support. By capturing analysis steps, parameters, software versions, and execution environments, these approaches make workflows less dependent on transient local installations or web services. This is increasingly important as multi-omics analysis relies on rapidly evolving reference databases, hosted analytical platforms, and AI systems whose behavior may change over time. For analysis involving such resources, reproducibility requires not only workflow sharing but also database snapshots, persistent identifiers, dates of access, archived parameters, and, where AI systems are used, documentation of model versions, prompts, settings, and relevant outputs. Publishing versioned workflows in registries such as the WorkflowHub^57^ enables discovery, reuse and long-term auditability. Effective multi-omics microbiome studies require global partnerships that share samples, expertise, and access to advanced instrumentation, ensuring equitable contribution and representation across regions^58^. Complementing these efforts, data repositories should also help capture underrepresented, region‑specific microbial functions that are often overlooked in global datasets, improving both taxonomic and functional resolution in multi‑omics integration^58,59^. Education and community coordination including tutorials, annotated workflows, reusable code, and teaching materials are foundational for broadening participation and ensuring that multi‑omics integration remains robust, reproducible, and widely applicable^59^.

Together, these practices form a blueprint for scalable, reproducible, and inclusive multi-omics integration in microbiome science. As the field moves toward coordinated efforts, such frameworks will be critical to ensure that multi-omics data are not only generated, but meaningfully interpreted and shared. Importantly, these best practices should be interpreted as a practical hierarchy rather than all-or-nothing standard. Some practices represent broadly applicable minimum requirements, which are (i) question-driven omics selection, with each omics layer justified by the biological question being addressed; (ii) sample-level linkage, with cross-omics datasets traceable to the same biological material or clearly justified paired samples; (iii) core metadata and quality control, with essential metadata, QC metrics, and workflow provenance reported; and (iv) reproducible analysis, with software versions, parameters, and execution environments documented. Others, including shared source material, harmonized protocols, and longitudinal sampling, represent recommended practices whose feasibility may depend on study scale, sample availability, and available resources. The aim is to make the recommendations suitable across different study contexts, including large, coordinated initiatives, smaller studies, and analyses of legacy datasets where ideal design is not always possible.

### Current efforts

To fully realize the synergistic potential of multi‑omics, the microbiome field must move beyond isolated method development and toward coordinated, benchmarked, and integration‑ready science. For this, the field must converge on shared best practices while strengthening international, coordinated collaboration. Ultimately, progress should be measured not by the volume of data generated but by the knowledge gained. Meaningful milestones will depend on reproducible functional patterns, quantitative assessment of the contribution of each omics layer, and generation of new hypotheses and experimentally supported links between molecular activity and phenotype and prioritization of the underlying components for future validation.

A foundation for this transition already exists. International consortia and community initiatives have produced standardized datasets, validated protocols and benchmarking frameworks that can be extended across omics layers and ecosystems. Examples for cross-ecosystem integration include the ongoing efforts for standardization of the key experimental parameters (e.g., pH, temperature, oxygen level, etc.) across the currently used in vitro colonic microbiota models to enable reproducible and comparable interpretation of data generated experimentally (INFOGUT) (https://infogut.eu/), while the Microbiome Biobanking Enabler project aims to facilitate the assembly of representative synthetic communities. Benchmarking consortia such as Critical Assessment of Metagenome Interpretation (CAMI) has strengthened the multi-omics ecosystem by providing community-driven evaluations of analytical tools^60^.

Community-driven initiatives such as the Genomic Standards Consortium^61^ are essential for advancing cross-study and cross-omics compatibility. The International Human Microbiome Consortium (IHMC) coordinated the development of standard operating procedures for sampling, sequencing, and metadata capture, now implemented and benchmarked in subsequent studies, e.g., in Costea et al^62^. Large-scale initiatives such as the Holomicrobiome Initiative and the Human Microbiome Project (HMP) with its integrative multi-omics extension (iHMP) demonstrate the feasibility of coordinated, longitudinal profiling across metagenomics, metatranscriptomics, metaproteomics, and metabolomics^63,64^. The Earth Microbiome Project (EMP) laid the groundwork for a globally coherent view of microbial diversity, and its standardized datasets now serve as a springboard for increasingly integrative multi‑omics analyses^65^. Finally, guidelines from consensus consortia such as Strengthening the Organization and Reporting of Microbiome Studies (STORMS)^66^ and the Standards for Technical Reporting in Environmental and host-Associated Microbiome Studies (STREAMS)^67^, and additionally, the recent community annotation guidelines for metaproteomics^68^ provide checklists for reporting study information, experimental design and analytical methods within a scientific manuscript on human and environmental microbiome research.

International initiatives such as “One Health” applications^3^ and longitudinal ecosystem perturbation studies^20^ have illustrated how coordinated multi‑omics designs can lead to mechanistic insight that would remain inaccessible to single‑omics approaches. Similarly, mechanistic frameworks such as the Adverse Outcome Pathway (AOP) framework provide an opportunity to strengthen causal inference by organizing multi-omics evidence into biologically coherent pathways linking microbiome perturbations to host phenotypes. Although primarily developed in toxicology, the incorporation of microbiota-mediated mechanisms into AOPs is beginning to emerge, offering new opportunities to translate microbiome multi-omics into regulatory science^69^. These initiatives signal a transition from isolated methodological development toward coordinated, benchmarked and integration-ready microbiome science. Among community-driven efforts aiming to advance integration across studies, omics, and ecosystems, the Metaproteomics Initiative^70^ is investigating improved integration with other omics methods, with the recently announced CAMPI‑Multi-omics study (https://metaproteomics.org/campi/). It exemplifies a two‑pronged strategy combining systematic comparison of existing datasets and workflows with the coordinated generation of new, best‑practice multi‑omics datasets.

We propose that the next phase of microbiome multi-omics should be organized around these efforts:

- **Tiered set of cross‑omics standards**. We propose that future microbiome multi-omics efforts distinguish between minimum reporting requirements necessary for reproducible integration and more advanced best-practice recommendations that may depend on study scale, ecosystem, or resource availability. Minimum requirements should include persistent sample-level identifiers across omics layers, standardized metadata schemas, transparent workflow provenance, and layer-specific quality-control reporting. Together, these elements provide the foundation for dataset traceability, reproducibility, and cross-study reuse.
- **Robust study design and validation**. More advanced recommendations may include longitudinal matched sampling, absolute quantification strategies, external validation across independent cohorts, and benchmarking using synthetic or controlled microbial communities. Although not universally feasible, these approaches can substantially strengthen the robustness, comparability, and generalizability of multi-omics integration studies.
- **Shared reference materials**. Circulate reference samples and synthetic communities across laboratories to quantify technical variance and validate harmonized protocols. Expanded access to living and cryopreserved microbiome libraries and representative synthetic communities will enable controlled, cross‑laboratory experiments that quantify technical variance and biological signal.
- **Advance and embrace emerging technologies that will accelerate multi-omics microbiome research**. Emerging long‑read and ultra‑deep sequencing platforms are enabling more complete genome assemblies, strain‑level tracking, and richer integration with metatranscriptomics and metagenomics. Next‑generation mass spectrometry platforms are poised to be transformative for metaproteomics and metabolomics.
- **Open benchmark datasets and tasks**. Coordinated release of cross‑ecosystem, cross‑omics benchmark datasets with defined tasks, evaluation metrics, and ground‑truth references will enable comparable integration methods and support robust model training and objective method evaluation.
- **Community Standard Operating Procedures (SOPs) for data merging**. Community‑driven benchmarking can produce consensus SOPs that specify what to merge, at which data level, and under which quality thresholds.
- **Aligned incentives**. Achieving this will require aligning partially conflicting incentives from funders, academic journals, and repositories to make complete metadata/provenance, transparent workflows, and dataset versioning routine.

## Conclusion

For investigators designing or interpreting microbiome multi-omics studies, the immediate priorities are to select omics layers according to the biological question, preserve sample-level linkage across molecular measurements, capture essential metadata and quality-control information, and document analytical workflows sufficiently to enable reproducible interpretation. These baseline practices provide a practical foundation for determining whether additional molecular layers contribute meaningful biological information rather than simply increasing data complexity. Delivering these practices consistently and at scale will, however, require supporting actions from consortia, repositories, funders, standards organizations, and other community-level initiatives. National and regional efforts organized around harmonization, benchmarking, shared reference materials, and open collaboration can provide the interoperable infrastructure needed to extend good practice beyond individual projects and enable reusable, AI-ready multi-omics resources. In this Perspective, we have therefore combined study-level recommendations for investigators across career stages and areas of omics expertise with community-level priorities that make their broader implementation possible. We also encourage early-career investigators to get involved in collaborative endeavors early on. This way, the new investigators benefit from the expertise of more experienced researchers, helping to avoid common pitfalls and to extend the usefulness of newly generated datasets far beyond the individual projects.
